# Assessing risk of bias in the meta-analysis of round 1 of the Health Care Innovation Awards

**DOI:** 10.1186/s13643-023-02409-9

**Published:** 2024-01-22

**Authors:** Kevin W. Smith, Nikki L. B. Freeman, Anupa Bir

**Affiliations:** RTI International, 307 Waverley Oaks Road, Suite 101, Waltham, MA 02452-8413 USA

**Keywords:** Risk of bias, Meta-regression, Medicare spending, Meta-analysis, Nonrandomized studies, Healthcare interventions

## Abstract

**Background:**

Systematic reviews of observational studies can be affected by biases that lead to under- or over-estimates of true intervention effects. Several tools have been reported in the literature that attempt to characterize potential bias. Our objective in this study was to determine the extent to which study-specific bias may have influenced intervention impacts on total costs of care (TCOC) in round 1 of the Health Care Innovation Awards.

**Methods:**

We reviewed 82 statistical evaluations of innovation impacts on Medicare TCOC. We developed five risk-of-bias measures and assessed their influence on TCOC impacts using meta-regression.

**Results:**

The majority of evaluations used propensity score matching to create their comparison groups. One third of the non-randomized interventions were judged to have some risk of biased effects due largely to the way they recruited their treatment groups, and 35% had some degree of covariate imbalance remaining after propensity score adjustments. However, in the multivariable analysis of TCOC effects, none of the bias threats we examined (comparison group construction method, risk of bias, or degree of covariate imbalance) had a major impact on the magnitude of HCIA1 innovation effects. Evaluations using propensity score weighting produced larger but imprecise savings effects compared to propensity score matching.

**Discussion:**

Our results suggest that it is unlikely that HCIA1 TCOC effect sizes were systematically affected by the types of bias we considered. Assessing the risk of bias based on specific study design features is likely to be more useful for identifying problematic characteristics than the subjective quality ratings used by existing risk tools.

## Background

As the number of systematic reviews of health care interventions has proliferated over the past decade, so too have concerns about potential biases that may exaggerate findings about average effect sizes in these reviews. To address these concerns, several risk-of-bias (ROB) instruments have been developed to measure risk for both individual studies [1] and for systematic reviews [2, 3] of observational (nonrandomized) studies. The degree of risk is typically classified as either low risk, high risk, or unclear for 5–7 key domains. Overall risk assessments are then created by combining the domain ratings. The overall risk is sometimes used to characterize the credibility of a body of evidence by examining whether mean treatment effect sizes differ by overall risk level.

Several problems have emerged in the application of these ROB instruments in meta-analyses of observational studies. First, many risk domains end up being classified as “unclear” due to a lack of detail in study reports. Second, inter-rater reliability of risk judgments for the same study is often less than ideal and investigators sometimes make inappropriate modifications to the tool domains [4]. Third, different ROB tools can produce conflicting conclusions about the overall ROB level for the same set of studies [5]. Fourth, there is little evidence that quality domains are actually associated with bias. Weighting results by quality scores have been found to be inadequate for removing bias [6, 7]. Finally, even when it can be assessed, a crude overall risk categorization is of limited usefulness because it does not quantify either the magnitude or the direction of bias in effect sizes. This has led some observers to recommend that ROB assessments should focus instead on key quantifiable aspects of study design rather than risk level categorization [8–10]. To improve the usefulness of meta-analysis, it would be more informative to measure specific study design features and then estimate the potential bias of these features using meta-regression.

Our overall objective in this secondary analysis was to assess the extent to which ROB may have affected the meta-analysis results from round 1 of the Health Care Innovation Awards (HCIA1; [11]). To make this assessment, we developed five ROB measures based on study design and analysis procedures. HCIA1 was in several respects an ideal laboratory in which to assess ROB: the initiative involved more than 100 evaluations, employed a wide range of healthcare innovations, examined a common outcome (quarterly Medicare spending per beneficiary), and used a variety of methods to select and balance comparison groups.

### Heath care innovation award sample

To be eligible for an award, applicants had to propose to implement new healthcare delivery service models that focused on improving coordination, efficiency, and quality of care. The first round of HCIA awards was made in July 2012 [11]. Awardees implemented a wide variety of innovation components including community health workers, medical home transformation, behavioral health programs, health information technology, workflow/process redesign, and telemedicine [12].

Innovations were classified as ambulatory care, post-acute care, or hospital-based. The Centers for Medicare and Medicaid Innovation (CMMI) contracted with independent research organizations (called Front Line Evaluators or FLEs) to conduct the outcome evaluations for each awardee. As the initiative’s meta-analysis evaluator, we had unique access to the results reported by all FLEs.

#### Evaluation report dispositions

**Fig. 1:**
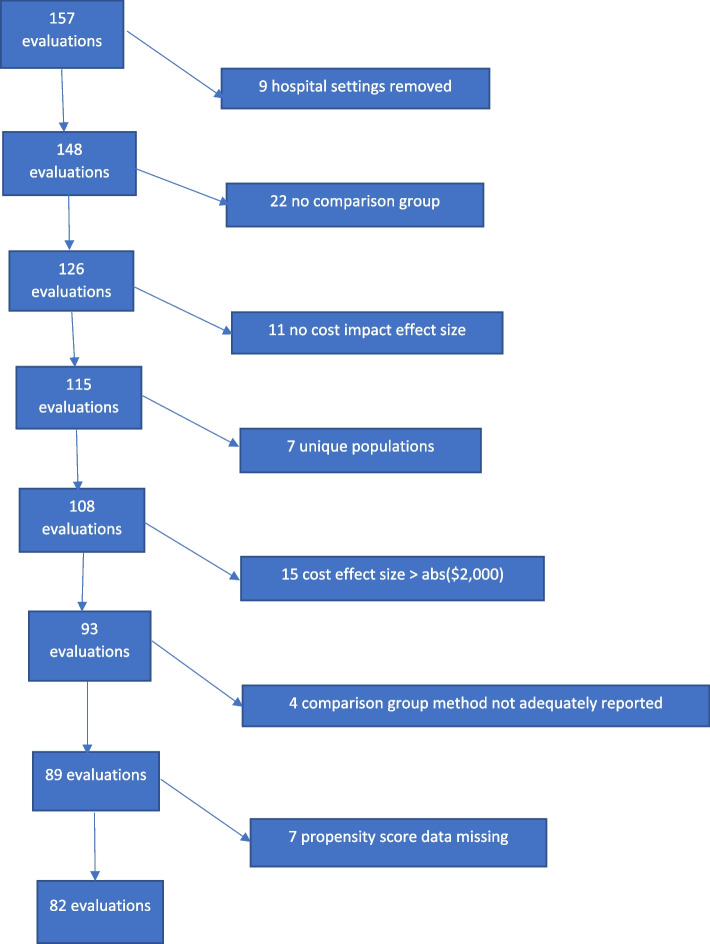
Disposition of HCIA1 evaluations and reasons for exclusion

We identified 157 unique evaluations submitted by FLEs by the end of the third year of the HCIA demonstration. The disposition of these evaluations and reasons for excluding evaluations from our analysis are displayed in Fig. 1. We focused on evaluations of ambulatory and post-acute care programs because these programs tended to offer similar types of primary care services and were most likely to be subject to the kinds of biases that we were interested in. We therefore eliminated programs in hospital settings and unique programs for special populations (e.g., palliative and hospice care, infants in intensive care, dental services for minority children). Other exclusions flagged evaluations with comparison group reporting inadequacies, and expenditure effect sizes that were either extreme values or missing. After applying the exclusion criteria, 82 evaluations were appropriate for our analysis.

## Methods

### Outcome measure

The outcome in our analysis was the estimated impact of a HCIA1 innovation on the Medicare Total Cost of Care (TCOC) per beneficiary per calendar quarter (PBPQ) over the 3-year period of project funding. Quarterly estimates were adopted as the standard reporting period to be used by all awardees. These impacts were estimated by FLEs using difference-in-difference regression models contrasting innovation and comparison groups while controlling for baseline differences in spending. Impact estimates may be negative (indicating savings relative to the comparison group) or positive (indicating comparative losses). We abstracted the quarterly impact estimates and their standard errors from final Year 3 Annual Reports submitted by the FLEs to provide the most comprehensive picture of overall intervention effects.

Our focus in this paper is on the impact of bias on TCOC effect sizes. Details about the range of TCOC effect sizes observed across all evaluations may be found in the project’s final report [11].

### Propensity model covariates

Nearly all FLEs used propensity scores (PSs) to construct comparison groups for innovations without a randomized control group [13, 14]. We recorded the types of covariates that FLEs used in their propensity score models, identifying the following characteristics:

1. Demographic characteristics (e.g., age, gender)
2. Prior costs (a baseline measure of beneficiary costs prior to the HCIA intervention)
3. Prior utilization (baseline measure of beneficiary health care utilization prior to the HCIA innovation)
4. Disease status (indicators for specific diseases like heart disease or type II diabetes)
5. Severity (an indicator for the severity of disease or a summative measure for risk such as a hierarchical condition code (HCC) risk score).

FLEs had very similar covariate measures because many of these items were drawn from Medicare claims data.

### Comparison group construction methods

We classified the comparison group construction method used by the FLEs into four broad categories:

1. Matching/PSM: Matching encompassed direct matching and matching using propensity scores (PSM). Direct matching entails matching individual treatment and comparison beneficiaries directly on covariate values. PSM involved either matching beneficiary PSs within a caliper range or by nearest neighbor matching.
2. Propensity score weighting (PSW): Weighting used PSs to differentially weight the comparison group beneficiaries. Methods included inverse propensity of treatment weights, standardized mortality ratios, and relative weights.
3. RCT: A few HCIA innovations were randomized controlled trials (RCTs) with a control group. For these analyses, the FLEs used the randomly assigned control group as the counterfactual.
4. Other non-PS: FLEs did not use PS matching or weighting to construct their comparison groups in several instances. These included comparison beneficiaries who received “care as usual” but were not enrolled in the HCIA1 innovation and one case in which comparisons were drawn from non-participating facilities within the same state.

### Propensity score model characteristics

Because of the widespread use of PSM, we examined whether matching was done with replacement, the ratio of treatment beneficiaries to comparison beneficiaries, and whether the FLE weighted multiple comparison matches downward (down-weighting) to make the effective sample size of the comparison group equal to that of the treatment group. Half of the PSM evaluations employed one-to-one matching, while the other half used one-to-many matching. Nearly all of the evaluations using one-to-many matching down-weighted the number of comparison group beneficiaries to match the number of innovation beneficiaries. Details about whether matching was done with or without replacement were not uniformly reported.

One of the most important reasons for using propensity scores is to improve the covariate balance between the treatment group and the comparison group. Covariate balancing involves minimizing, on average, differences between the treatment and comparison groups on a set of observed covariates. For each evaluation using propensity scores, we recorded the number of covariates used in the FLE’s PS model and the number of covariates that remained unbalanced after matching or weighting and calculated the percentage of covariates that remained unbalanced at the 0.1 absolute standardized mean difference threshold [15].

### Risk of bias assessment

Any nonrandomized comparison poses a risk of bias when estimating intervention effects. The bias may be favorable (making the intervention appear to be more effective than it really was) or unfavorable (yielding intervention effects that are too small). For this report, we conducted a comprehensive review of the potential for biased effect estimates among the HCIA interventions.

To assess the risk of bias, we reviewed FLE’s Third Annual Reports and Addendums following no-cost extension periods for descriptions of how treatment and comparison groups were formed. Most ambulatory care programs shared a similar approach to group design. Treatment groups were assembled by establishing basic eligibility criteria for the intervention and relying on providers, facilities, or third parties to identify and recruit patients. In some cases, patients had to actively enroll or to comply with a set of conditions to be considered a treatment group participant. A finder file of “enrollees” or “participants” was generated from facility records to be linked to medical claims. Comparison group beneficiaries, on the other hand, were Medicare or Medicaid beneficiaries with similar diagnoses and hospitalization patterns drawn from neighboring geographic areas. The size of potential comparison pools was frequently very large, and FLEs relied heavily on propensity score matching to identify a much smaller group of comparison beneficiaries.

We initially attempted to use the ROBINS-I tool for assessing the risk of bias in non-randomized studies of interventions [1] but abandoned that effort when it became clear that the evaluation reports did not contain sufficient detail to code all of the ROBINS-I domains. Instead, we developed a set of specific bias threats. These included interventions in which the treatment group consisted of volunteers, patients who were required to actively enroll in programs, group status dependent on meeting participation or compliance criteria (such as attending a minimum number of sessions), or providers choosing patients they felt were the most “suitable” for their intervention. We also noted all cases flagged by FLEs as potential bias problems. If we felt that sufficient risk was present and that the direction was most likely in a favorable direction, we coded the evaluation as probable favorable bias (PFB). When the direction of the bias was unclear, we coded it simply as the risk of bias.

We coded the following 5 ROB indicators and one additional feature of the innovations:

- Potential favorable bias (PFB). PFB flagged evaluations in which we suspected that the recruitment process may have produced a favorable bias in the TCOC effects. The PFB effect was expected to be negative, indicating greater savings in expenditures.
- Risk of bias: These were evaluations suspected of bias, but for which the direction of bias was unclear. These problematic evaluations were frequently flagged by FLEs in their reports.
- PS Weighting: This binary code designated evaluations in which TCOC effects were estimated using PSW. All other designs (PSM, randomization, geographic region) served as the reference group for this contrast.
- Percent unbalanced covariates: We computed the percent of PS covariates that remained unbalanced after matching or weighting. We expected that this effect would be close to zero because imbalance could produce either positive or negative outcome effects.
- Barriers to recruitment: Some evaluations reported that they experienced problems recruiting patients for treatment. These evaluations were expected to be prone to favorable selection because they frequently liberalized their recruitment protocols in an attempt to draw more beneficiaries into their treatment group.
- New innovation. A previous analysis [12] found that HCIA1 TCOC effects were more likely to show comparative expenditure losses when awardees implemented new programs that they did not have previous experience with. This variable was included to remove some of the known variance in TCOC effects that was not related to the risk of bias.

Of the five ROB indicators, the PS weighting variable assesses design bias due to the way that comparison groups were formed. The other 4 measures all flag potential selection or confounder bias attributable to awardee recruitment procedures, lack of covariate balance, or recruitment problems.

ROB indicators were initially coded by junior analysts; all coding was reviewed by the senior author. To the extent possible, we followed the PRISMA abstraction guidelines for systematic reviews and meta-analyses [16].

### Meta-regression analysis

We used the *I*^2^ statistic [17] to assess heterogeneity in the data. *I*^2^ estimates the percentage of the total effect size variance attributable to between-intervention differences. To determine whether aspects of the comparison group construction had any systematic impacts on TCOC effects, we estimated a meta-regression model for the 82 evaluations in our sample using the weighted regression procedure in R. Results for each awardee were weighted by the inverse of the effect size estimate. The explanatory variables in the regression model consisted of the 6 ROB indicators described in the previous section.

## Results

### Awardee characteristics

Of the awardees in our analysis sample, 76% were ambulatory care programs, 24% were post-acute care programs, 80% had interventions that directly touched patients (as opposed to indirect training or electronic health record enhancement), 11% were for-profit health care organizations, and half served at least some patients in rural areas. Further details regarding the awardees may be found in Bir et al. [11].

### Propensity model and risk of bias characteristics

The majority of evaluations (77%) used matching, usually PSM, to construct a comparison group. Propensity score weighting (PSW) was utilized in 10% of the evaluations and RCTs comprised 6% of the evaluations. The remaining 8% relied on existing groups for comparisons.

All evaluations that used propensity score methods to construct their comparison group included basic demographic information in their propensity score models. Eighty-one percent of the PS models included prior utilization measures and 66% had prior beneficiary spending. Severity measures were included in 78% of the models and disease-specific indicators in 37%.

The number of covariates that FLEs used in their propensity score models ranged from 2 to 153; the median number of PS covariates was 14. Just over half of the evaluations achieved balance at the 0.1 standardized difference threshold on all the covariates included in their models. The mean percentage of unbalanced covariates was 9.6%. In 4 evaluations, the groups remained unbalanced on more than half of the PS covariates even after matching.

The prevalence of the other risk of bias measures is summarized in Table 1. We assigned PFB status to 27% of the evaluations; another 6% were classified as ROB of indeterminate direction. Twenty-six percent of the evaluations experienced barriers to recruitment and 30% of the innovations were new programs for their awardees.

**Table 1:** Meta-regression results for the impact of risk of bias indicators on TCOC effect size in dollars per beneficiary per quarter (*N* = 82)

ROB variable	Mean or percent	Coefficient estimate ($)	Standard error	*p* value
Intercept	–-	43.8	48.5	0.37
PS-weighting method (PSW)	10.1	− 328.9	613.6	0.59
Potential favorable bias (PFB)	27.0	− 59.3	63.4	0.35
Risk of bias, direction unclear	5.6	− 21.9	91.0	0.81
Percent unbalanced covariates	9.6	− 0.89	1.71	0.61
Barriers to enrollment	25.8	− 78.8	97.0	0.42
New innovation	30.3	156.8	70.1	0.03

### Meta-regression results

The mean TCOC effect sizes in this sample ranged from - $1443 per quarter to + $1927 per quarter, with a mean effect size of $19 (SE = $21). The *I*^2^ heterogeneity statistic for our evaluations was 82.9%. One benchmark for interpreting *I*^2^ values is that 25% indicates low heterogeneity among effect sizes, 50% indicates moderate heterogeneity, and 75% or more indicates high heterogeneity [17]. Given the high heterogeneity level among TCOC effect sizes in our data, we employed meta-regression in an attempt to explain this variation based on study characteristics.

Descriptive statistics and the results from our meta-regression are shown in Table 1. As hypothesized, PFB was associated with TCOC savings (negative TCOC effects) of $59 (SE = $63) dollars per beneficiary per quarter. Evaluations that were classified as being at risk for bias of indeterminate direction were also associated with TCOC savings of $22 (SE = $91) PBPQ as were barriers to enrollment ($79 comparative savings (SE = $97). However, these savings effects were of relatively small magnitude and statistically insignificant.

The impact of PS weighting vs matching was quite large (-$329 PBPQ), but this is a very imprecise estimate due to the small number of evaluations using weighting. As expected, the impact of covariate imbalance was close to zero. Like our previous meta-regressions, newly implemented innovations were associated with significant dissavings ($157 PBPQ, SE = $70).

The large PSW effect may have been influenced by the fact that all of the PS-weighted comparison groups came from post-acute care setting innovations. Post-acute setting interventions have more variable effect sizes because of higher spending levels compared to ambulatory care settings. Furthermore, the post-acute programs were smaller, on average, than the ambulatory care setting programs; this means that their coefficients were less precise and had smaller weights in the meta-regression.

## Discussion

The results from this meta-regression are reassuring because they indicate that the bias-related variables we considered had only negligible effects on TCOC. This suggests that the initiative’s difference-in-difference effects are unlikely to be contaminated by systematic biases associated with the way comparison groups were created, and we can be more confident that the overall meta-analytic results are not being dominated by a handful of potentially biased results.

Some evidence for the validity of our bias coding is provided by examining extreme estimates. Of 19 difference-in-difference effects that were later determined to be TCOC outliers, more than half had been labeled PFB.

One of the biggest challenges we encountered in making risk assessments was the lack of detail in the FLE reports about how “enrollees” and “participants” were defined for the treatment group finder files. The enrollment process was often a mystery. Rates of pre-screening, refusal to participate, and opting out were not described. The extent to which providers deliberately selected certain types of patients for their programs was rarely mentioned. As a result, it is possible that we have underreported the true risk because the incriminating information was not given in the annual reports that would have permitted us to make an accurate classification. Another complication is that some awardees changed their protocols over time. This seemed to be more common among awardees who were having patient recruitment problems and began relaxing or eliminating enrollment criteria to increase the size of their treatment groups.

Our measure of covariate balance was less than ideal. While the percentage of unbalanced covariates provides a crude assessment of group comparability, it can be affected by both the type and number of covariates selected for a particular study. A better measure of overall comparability would be the point-biserial correlation between treatment status and the weighted propensity score. Smaller correlations imply less bias given the observed covariates. Other potential indicators that we were unable to discern were the extent of sample attrition and whether evaluators accounted for the lack of common support in their analysis samples.

An unresolved issue is the degree to which PSM and PSW should be expected to produce the same outcome results. We found that PSW evaluations yielded considerably larger TCOC savings estimates on average, but these results were imprecise due to the small number of evaluations that employed PSW. This may have occurred because readily available software made it relatively easy for FLEs to automate the PSM matching process. However, PSM comparison groups are a smaller subset of the subjects used by PSW with different survey weights. Fullerton et al. [18] found that different matching methods (especially exact or coarsened matching) can yield different intervention effects than propensity-based matches for the same data set.

There are currently two main approaches to adjusting for bias in meta-analysis [19]. One method requires a panel of experts to make judgments regarding the magnitude and certainty intervals for a set of internal and external sources of bias. The results for individual studies are then weighted by the certainty estimates to derive an overall treatment effect [20, 21]. The alternative, which we favor, is to employ meta-regression to adjust for specific types of bias [22]. The meta-regression results are weighted by the precision of the estimates for each study.

Our experience attempting to code the ROBINS-I instrument led us to develop ROB measures for specific aspects of group comparability, patient recruitment, and comparison group methodology. Design features like these are easier to code and permit investigators to directly estimate the influence of a specific feature on outcome effect sizes. We believe that compiling a compendium of these features will be more informative for pinpointing specific bias threats and their magnitude than the current practice of making subjective assessments of overall study quality.

## Data Availability

The data used in this study are not part of a registry. Abstracted information can be made available on reasonable request with the permission from the Centers for Medicare and Medicaid Innovation.
